# Colonization of Microplastics by Different Strains of *Pseudomonas Syringae* Increases Ice-Nucleation Activity

**DOI:** 10.1021/acs.est.5c18769

**Published:** 2026-03-11

**Authors:** Carrie Carpenter, Kelsey Kern, Regina Hanlon, Boris A. Vinatzer, David G. Schmale, Hosein Foroutan

**Affiliations:** † School of Plant and Environmental Sciences, Virginia Tech, Blacksburg, Virginia 24061, United States; ‡ Department of Biological Sciences, Virginia Tech, Blacksburg, Virginia 24061, United States; § Department of Civil and Environmental Engineering, Virginia Tech, Blacksburg, Virginia 24061, United States

**Keywords:** ice nucleation, microplastics, biofilm, immersion freezing

## Abstract

Microplastics (MPs) are increasingly detected throughout the atmosphere, raising questions about their persistence and influence on cloud-relevant ice nucleation processes. Recent studies suggest that MPs may act as ice-nucleating particles (INPs), potentially enhanced by biological colonization. Here, we quantify the ice nucleation activity (INA) of polystyrene (PS) and polyethylene (PE) MPs before and after surface aging and microbial colonization. Strains of *Pseudomonas syringae*, spanning a range of INA, were cultured onto pristine and aged 100 μm PS and PE MPs. Uncolonized MPs (0.5 to 100 μm PS; 100 μm PE) exhibited INA, with median freezing temperatures ranging from −21.0 °C to −23.8 °C. Hydrothermal and photooxidation exposure, produced small, statistically insignificant increases in freezing temperature. PE MPs nucleated ice at higher temperatures than PS MPs, while the size of MPs did not appear to impact mean freezing temperatures. However, biofilm colonization increased median freezing temperatures by ∼6.5 °C (*p* < 0.0001) and enhanced INA relative to noncolonized MPs and cells alone. These results indicate that atmospherically relevant MPs modified by aging and microbial growth exhibit elevated INA, highlighting an under represented pathway by which MPs may influence cloud microphysics.

## Graphical abstract

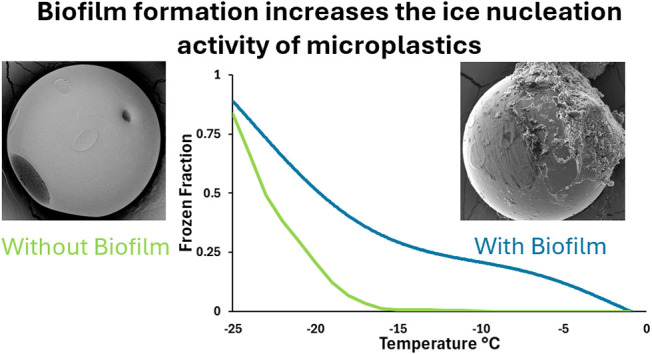

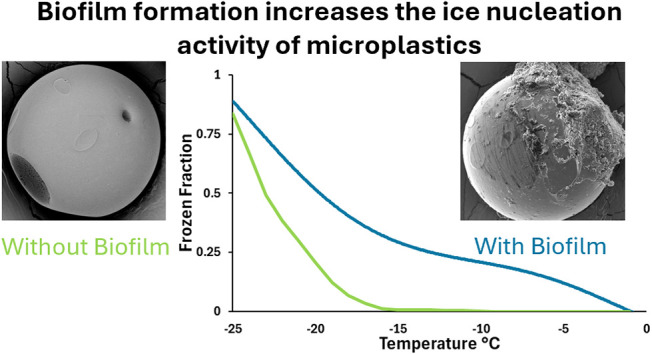

## Introduction

Microplastics (MPs), defined as plastic particles smaller than 5 mm, are ubiquitous throughout the biosphere, including the atmosphere.^1^ Recently, MPs have been found in remote and untouched areas,^2–6^ cloudwater,^7,8^ hailstones,^9^ and other forms of precipitation.^10–12^ These findings suggest MPs may act as ice-nucleating particles (INPs)^13,14^ —particles that can catalyze the freezing of water at higher temperatures. The occurrence and types of INPs are important to study as their presence in clouds changes the ratio of liquid to frozen water, which in turn^14^ affects Earth’s radiation balance and overall temperature.^15^ Recent modeling studies suggest that MPs may represent up to 40% of INPs in tropical regions and up to 20% under cirrus conditions in East Antarctic regions.^16^ However, this estimate is based solely on urban road emission sources, suggesting that MP particles could potentially play a larger role when all emission sources are considered.^17^ New information is needed to investigate the role of MPs in ice-nucleation processes in the atmosphere, and their potential impact on cloud formation and radiation budgets.^16^

Recent studies have examined the ice nucleation activity (INA) of MPs of varying shapes, sizes, and types including polyethylene (PE), low-density polyethylene (LDPE), polyvinyl chloride (PVC), polystyrene (PS), polypropylene (PP), polyethylene terephthalate (PET),^13,18–20^ and synthetic microfibers from clothing textiles (CT).^21^ Once MPs enter the atmosphere, they are subjected to weathering or aging processes such as photooxidation via UV exposure, which alters their surface chemistry, therefore modifying how the MP interacts with its surrounding environment.^22^ A few studies have examined how different forms of aging affect the ice nucleation activity of MPs, but their findings are inconsistent.^18–20^ While these studies demonstrate that both pristine and aged MPs, across various polymer types and sizes, can function as ice nucleators, the role of biological material on their surfaces remains unclear.

Microorganisms are known to colonize MPs, and the community of microorganisms associated with MPs in a given environment is known as the plastisphere.^23^ Once attached, microbes can alter the surface properties of MPs including hydrophobicity, density, roughness, size, and functional groups.^24^ These modified surface characteristics have the potential to influence their transport.^25^ Ice nucleation activity (INA) is often linked to associations with biological ice nucleators.^26,27^ For example, Teska et al. showed that biological material on the surface of textiles was contributing to INA.^21^ This underscores the importance of studying the impacts of biofilm formation on MP surfaces related to their ice nucleation capability.

The bacterium *Pseudomonas syringae* is a model organism for the study of biological ice nucleation processes,^28–31^ and some strains of *P. syringae* are known to colonize and form biofilms on MPs.^32^ In natural systems, biofilms form on MPs while they reside in soil, water, or vegetation surfaces, and biofilm-coated particles may later become airborne through mechanical disturbance or aerosolization. We hypothesized that environmental aging processes and biofilm formation increase the INA of MPs. To test these hypotheses, the *P. syringae* strain TLP2 (potato leaf pathogen isolate) either with INA (ice+) or deficient INA (ice-) due to deletion of the *InaZ* gene,^33^ were cultured on pristine and aged PS and PE MPs, each 100 μm in diameter. The specific objectives of our work were to (1) age MPs using UV light or hydrothermal treatment; (2) grow *P. syringae* biofilms on the surfaces of aged and pristine PE and PS microspheres; (3) subject MP treatments to a freezing droplet assay;^34^ and (4) assess how aging and biofilm formation influence the INA of MPs. These results have important implications for incorporating environmentally relevant MP characteristics into atmospheric models of INPs.

## Materials and Methods

### Selection of MPs

A list of the MPs and treatments is provided in Table 1. Polystyrene (PS) microspheres sized 0.5 μm, 2 μm, 6 μm, and 10 μm were obtained from Polysciences (Warrington, PA, USA); 1 μm PS microspheres were obtained from Thermo Fisher Scientific (Walthom, MA, USA); and 100 μm PE and PS microspheres were obtained from Abvigen (Skillman, New Jersey, USA). All microspheres were received in a solution of deionized (DI) water containing approximately 0.05% NaN_3_ and were considered to be pristine. Occasional surface pits/craters on 100 μm beads are manufacturing artifacts known for suspension/emulsion polymerized microspheres. PS and PE polymers were selected because both have been detected in atmospheric samples^35^ and, in preliminary testing, supported consistent biofilm attachment for our bacterial strains, enabling reproducible surface colonization.

**Table 1:** Description of Microplastics Used in the Experiment^a^

polymer	size	origin	surface modifications	treatment
PS	0.5 μm (±0.05 μm)	polysciences, Inc. (17,152-10)	N/A	pristine, hydrothermally aged
PS	1 μm (±0.023 μm)	ThermoFisher (F8823)	carboxylate (CaPS)	pristine, hydrothermally aged
PS	1 μm (±0.026 μm)	ThermoFisher (F8852)	sulfate (SuPS)	pristine, hydrothermally aged
PS	1 μm (±0.032 μm)	ThermoFisher (F8765)	amine (AmPS)	pristine, hydrothermally aged
PS	2 μm (±0.2 μm)	polysciences, Inc. (24,172-5)	biotin (PS_Bio)	pristine, hydrothermally aged
PS	2 μm (±0.2 μm)	polysciences, Inc. (24,159-5)	streptavidin (PS_strept)	pristine, hydrothermally aged
PS	6 μm (±0.6 μm)	polysciences, Inc. (17,156-2)	N/A	hydrothermally aged
PS	10 μm (±1 μm)	polysciences, Inc. (18,140-2)	N/A	hydrothermally aged
PS	100 μm (±10 μm)	abvigen (ABWR-21-9000)	ice+ and ice− biofilms	pristine, UV aged
PE	100 μm (±10 μm)	abvigen (ABPER-10000)	ice+ and ice− biofilms	pristine, UV aged

a Information includes polymer type, size, origin, surface modification, and treatment.

### Aging of MPs

Both UV and hydrothermal aging treatments were applied to MPs to simulate weathering conditions. For UV-aging, 100 μm PS and PE microspheres were first suspended at six MPs per microliter in 10 mL 0.2 μm-filtered, autoclaved DI water in a sterilized 50 mL beaker to create a stock suspension for irradiation. Each beaker was covered with a 60 × 45 mm glass slide (TedPella) and placed inside a custom-built weathering chamber equipped with a 15 W UVC lamp (260–280 nm wavelength) for three consecutive days.^36^ This duration was selected based on prior studies demonstrating that 72 h of UVC irradiation at comparable wavelengths induces measurable photo-oxidative aging and surface alteration in polymeric microplastics (e.g., oxidation, cracking, increased surface roughness), making it a widely used accelerated aging protocol for laboratory simulation.^36,37^ This UVC-based treatment represents an upper-bound, accelerated aging scenario and is not intended to replicate natural atmospheric exposure, which is dominated by UV-A and UV-B wavelengths.

At 24 and 48 h, beakers were covered with sterilized foil and placed into a sonicator for 10 min at 20 kHz for mixing to ensure even treatment of the sample. After 72 h, the beakers were removed from the chamber, covered with parafilm and sterile foil, and stored in the dark at 4 °C until used. Hydrothermal aging was performed by autoclaving 0.5–10 μm PS microspheres at 240 °C for 15 min under a gravity cycle. This process exposed the particles to both elevated temperature and pressure to mimic thermal and mechanical environmental stress.

All pristine MPs were checked for contamination by spread plating on R2A media and used as received in DI water containing ∼0.05% NaN_3_. Pristine MPs were not subjected to any aging treatment. Surface modifications resulting from both UV and hydrothermal aging were verified using scanning electron microscopy (SEM) by comparing aged and pristine MPs (Figure S1).

### Growing a Biofilm on MPs

*P. syringae* TLP2 wild type (ice+) and its isogenic mutant (ice−)^33^ were separately inoculated with 100 μm MPs. Each *P. syringae* strain had a single colony inoculated into 4 mL of King’s B broth media [0.9 g Potassium Phosphate Monobasic, Anhydrous, (MP Biomedicals); 9.0 g Bacto Proteose Peptone, (Gibco Thermo Fisher); 9 g agar (ACROS organics); 6 mL Glycerol 99+%, extra pure, (Thermo Scientific)] and incubated on a shaker (Excella E24 Incubator Shaker Series, New Brunswick Scientific) at 28 °C and 220 RPM overnight.

The optical density at 600 nm (OD_600_) was measured using 600 μL aliquots of ice+ and ice− cultures with a WPA Biowave (CO8000 cell density meter, Avantor, Radnor, PA, USA). Cultures were diluted to an OD_600_ of 0.15, which represented ∼1.6 × 10^6^ of cells determined by growth curves prior to treatment. Three-milliliter aliquots of the diluted *P. syringae* cultures were then placed into four 15 mL culture tubes (17 × 100 mm, Olympic Plastics) each. One milliliter of 100 μm UV-treated PS (PSUV), UV-treated PE (PEUV), pristine PS, and pristine PE was added to the respective tubes, resulting in working concentrations of 2 MPs/μL for UV-treated and 3 MPs/μL for pristine in a total volume of 4 mL. To achieve close to accurate concentrations, bottles and flasks with suspended MPs were continuously gently shaken while taking out aliquots, ensuring the MPs were evenly dispersed within the solution. The tubes containing *P. syringae* cultures and 100 μm MPs were then placed into the shaker incubator at 28 °C and 220 rpm for 24 h to allow initial microbial attachment and early biofilm formation on the MP surfaces. Cultures were filtered through a 70 μm filter (Greiner Bio-One EASYstrainer) to collect and retain the 100 μm MPs, and tubes were rinsed with 3 mL of 1X PBS and filtered before being resuspended in 4 mL of fresh KB broth. Tubes were returned to the incubator at 28 °C, 220 RPM. This medium-refreshment step was repeated after 48 h to maintain nutrient availability and promote continued biofilm development, before harvesting MPs with established biofilms at 72 h.

### Harvesting MPs with Biofilms

After 72 h, all tubes were taken out of the incubator and processed for imaging, sonication, and a freezing droplet assay. Cultures were filtered through the 70 μm filters, including 6 mL of 1X PBS that was used to rinse the tubes. To ensure concentrations stayed the same, excess liquid held in the filters via capillary action was removed from the bottom of the filters, using a 1 mL micropipette before resuspension. The cultured MPs were then resuspended in 1.5 mL of 0.22 μm filtered, autoclaved DI water. Five hundred microliters were then taken from the 1.5 mL and placed into a separate centrifuge tube. The remaining 1 mL suspended in water was filtered again before being resuspended in 1 mL PBS. From the 500 μL tube, 100 μL was added to 300 μL of 2.5% glutaraldehyde (50 wt % in H_2_O, Millipore) which was then placed on a rocker at 4 °C to allow cells to fix overnight. The remaining 400 μL of suspension in water was placed at 4 °C until use.

The 1 mL PBS fraction was then sonicated at 20 kHz for 10 min to dislodge the biofilm from the MPs. These sonicates were then subjected to a serial dilution before 50 μL was spread plated in triplicate on KB agar plates (15 × 100 mm). Plates were incubated at 28 °C for 48 h before colony forming unit (CFU) counts were recorded.

### Freezing Droplet Assay

Modified from Vali et al.,^34^ 12 μL droplets were placed into a 96-well assay plate, made of nontreated polystyrene (Costar) and were floated on a Lauda cooling bath (Alpha RA12).^38,39^ To visualize droplet freezing, fluorescein disodium salt dye (ACROS) was used at a final concentration of 250 ppm in all sample preparations, including water controls. This solvatochromic compound changes color depending on the solvent environment, transitioning from green to orange upon freezing (Figure S2).^40,41^ We also conducted analyses to ensure the addition of fluorescein did not impact the freezing results (Figure S3). One-hundred-micron PE, PS, PEUV, and PSUV without biofilm were prepared in water at two concentrations: 6 MPs/μL and 0.6 MPs/μL. All other pristine and hydrothermally aged microspheres were prepared in water with concentrations of 6000 MPs/μL, 600 MPs/μL, and 60 MPs/μL. The MPs with biofilm formation had a final concentration of 8 MPs/μL for pristine and ∼5.3 MPs/μL for UV-treated after filtration and resuspension. Although the 100 μm microspheres and their biofilm constituents were at a lower concentration due to having less particles present in the stock solution, each 100 μm MP treatment contributed more geometric surface area, so the total available surface area in the droplet was not reduced proportionally. Samples with positive (*P. syringae* ice+ cells in pure water) and negative (pure water) controls were loaded into 96-well plates and floated on the cooling bath starting at 0 °C. Replicate measurements for each treatment were conducted within individual 96-well plates, and no interplate calibration was applied. Treatments were not fully randomized across plates due to biological constraints associated with microbial colonization, which required colonized microplastics to be prepared and measured concurrently. Accordingly, data interpretation focuses on relative differences among treatments measured within the same experimental runs. The temperature was decreased by 1 °C and held at each temperature for 2 min until −25 °C (instrument limit) was reached. At the end of the 2 min hold for each decrease in temperature, frozen droplets (orange) were counted and recorded before continuing to decrease the temperature. This method is not without limitations as the temperature range of the droplet-freezing assay was limited to −25 °C due to the operational limits of the cold-stage apparatus. As a result, freezing behavior below this temperature was not resolved, and the data primarily capture the onset and median portions of the frozen-fraction curves. Median freezing temperatures (*T*_50_) were therefore used for comparison among treatments within this measured range.

To determine the cumulative frozen fraction, *F*(*T*), the following equation was used *F*(*T*) = *n*(*T*)/*N*, where *n*(*T*) is equal to the number of frozen droplets at a given temperature and *N* is the total number of droplets for each sample. A frozen fraction curve was created from averaging across all trials for each sample. Using frozen fraction, the cumulative density of active nucleation sites per volume *K*(*T*) was calculated using

1 K(T)=−ln(1−F(T))/V

where *V* is the volume of each droplet (0.012 mL). To account for background freezing of our setup, the cumulative density of active nucleation sites associated with the background control water *K*_background_ was determined in each experiment and subtracted out. i.e, *K*_sample_(*T*) = *K*(*T*) – *K*_background_(*T*)*.* Therefore, *K*_sample_(*T*) is the cumulative density of active nucleation sites per volume pertaining only to the particles. This value was further normalized to the mass of MPs using eq 2

2 $$n_{m}\left(T\right)=K_{sample}\left(T\right)/C$$

where *n*_m_(*T*) is the number of active sites per mass of MPs (g^–1^) and *C* is the concentration of suspension in g/mL.

At temperatures where background freezing exceeded the sample signal, resulting in negative *K*_sample_(*T*), we conservatively assigned *K*_sample_(*T*)—*K*_background_(*T*). Shaded regions reflect uncertainty, spanning from a small positive lower bound to the background value, indicating that the sample signal could not be reliably distinguished from background freezing under these conditions. All ice-nucleation data were normalized by sample mass to maintain consistency between polymeric particles and biofilm-bearing samples. This convention aligns with biological INA studies that report activity per unit biomass. Approximate surface-area scaling, based on particle size distributions, yielded the same relative trends, confirming that the main conclusions are not dependent on the chosen normalization basis. Direct surface-area normalization for biofilm-coated particles was not possible due to irregular and variable morphology. All statistical analyses were done in Igor Pro9 using standardized *t* tests on median freezing temperatures.

### Imaging Biofilms on MPs

To verify the formation of biofilm growth on the plastic polymers, each treatment was imaged using scanning electron microscopy (SEM), see Figure 1. Cultured MPs were rinsed with 3 mL of 1X PBS and placed into 2.5% glutaraldehyde solution (Millipore) rocking overnight to stabilize and fix the cells in place. Samples were rinsed again with PBS before being subjected to an ethanol dehydration series (30%, 50%, 70%, 80%, 90%, 100% x2) incubating within each solution for 5 min. After MPs were air-dried in a laminar flow hood, they were mounted on 1.25 mm SEM mounts and coated with a 10 nm thick layer of platinum palladium using a Cressington 208HR Sputter (Leica, Wetzlar, Germany). Samples were then imaged using a JEOL IT500 SEM at 5.0 kV (JEOL, Peabody, Massachusetts, USA).

**Figure 1:**
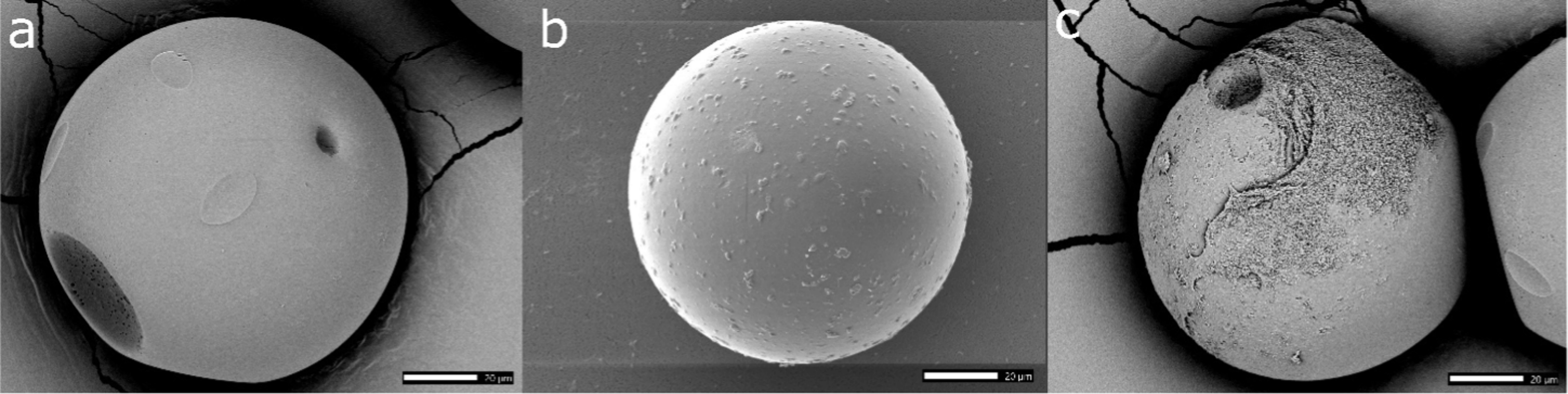
Scanning electron microscope (SEM) images of PE MPs subjected to three treatments: (a) pristine 100 μm PE, (b) UV-aged 100 μm PE, and (c) *P. syringae* biofilm on 100 μm PE. Each image is shown with a 20 μm scale bar.

## Results

### Freezing of Pristine and Aged Microplastics

The INA of pristine and aged MPs is displayed as boxplots in Figure 2. Aging treatments altered the temperature at which 50% of droplets froze, with the direction and magnitude of change depending on particle type. Specifically, AmPS 1 μm, SuPS 1 μm, CaPS 1 μm, PS Bio 2 μm, and PS strept 2 μm exhibited slight increases in median freezing temperature (up to +0.5 °C), while PS 0.5 μm, PS 100 μm, and PE 100 μm showed decreases (−0.1 °C, −0.7 °C, and −1.7 °C, respectively). However, when grouped by polymer type, no statistically significant difference was observed in INA between pristine and aged PS or PE particles (*p* > 0.05). In contrast, polymer identity significantly influenced INA overall, with PE freezing nearly 2 °C warmer than PS (*p* = 0.015). Regarding the number of active nucleation sites, results were mixed: PS 0.5 μm, AmPS 1 μm, PS bio 2 μm, and PS strept 2 μm showed no change and/or an increase in activity between −10 °C and −16 °C. After surpassing −17 °C, most MPs showed reduced activity, while SuPS 1 μm showed an increase in activity. However, samples were not always consistent with a few having fluctuations between an increase and a decrease in activity as the temperature dropped (Table S1).

**Figure 2:**
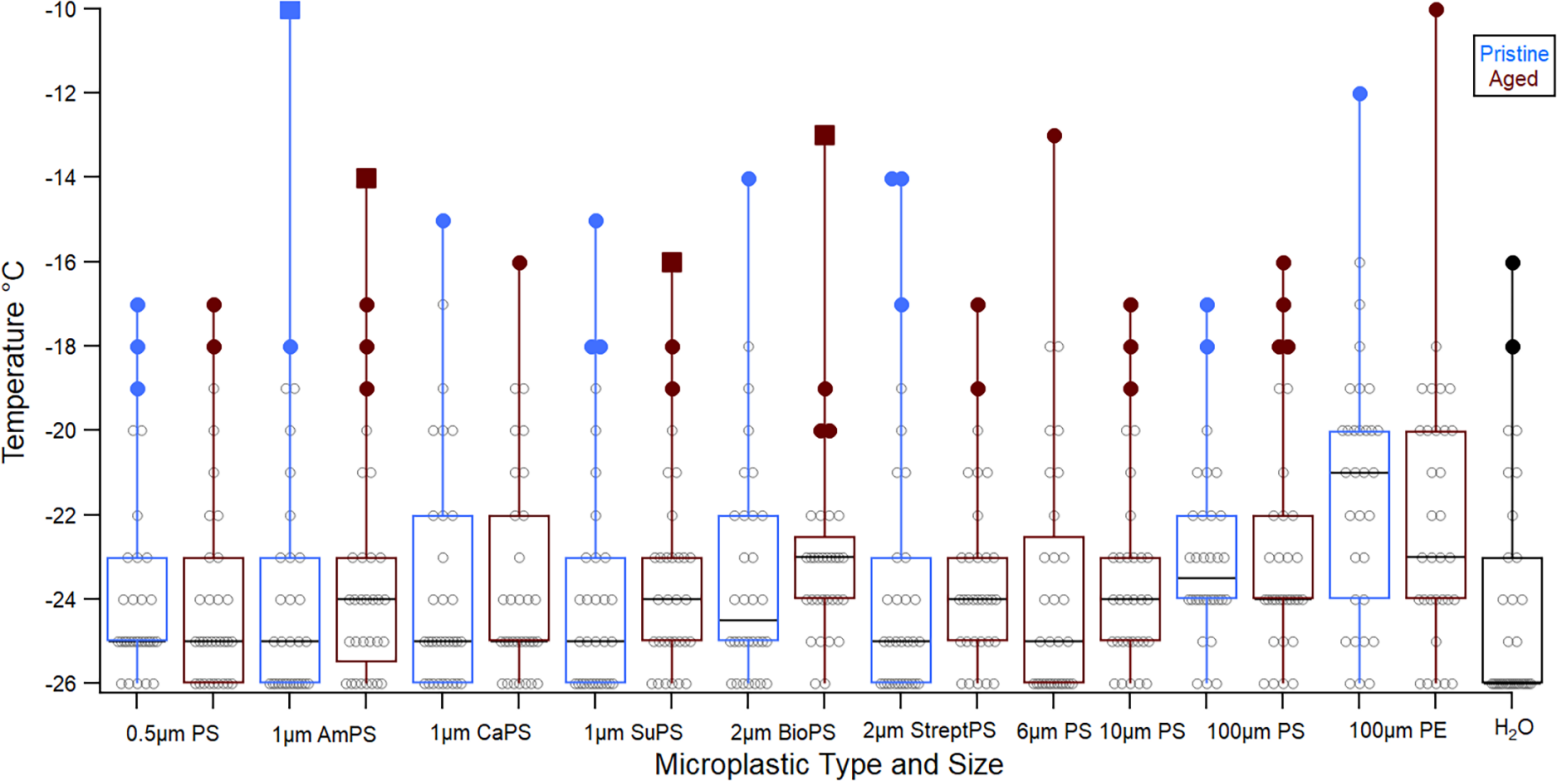
Boxplots showing the freezing temperature distributions of microplastic samples (as listed in Table 1) before and after aging treatments. Sample types are shown along the *x*-axis, and freezing temperature is plotted on the *y*-axis. Freezing temperature distributions for pristine and aged microplastics of the same type and size are shown in blue and brown, respectively. Each boxplot includes data from at least three replicates. The box bounds indicate the interquartile range (25th–75th percentiles), with the median freezing temperature (*T*_50_) shown as a black horizontal line. Filled circles represent outliers, and filled boxes indicate far outliers. Whiskers extend to the 10th and 90th percentiles. The black box on the far right represents the water control. All MPs were tested at the same concentration except for 100 μm microspheres due to manufacturer-supplied stock limitations; however, their substantially larger geometric surface area minimizes the functional impact of this difference. Note: due to limitations of the cooling bath, droplets that remained unfrozen at −25 °C were recorded as freezing at −26 °C to reflect the average behavior of the trials.

### Impact of Microplastic Size on Ice Nucleation

The relationship between size and median freezing temperature is shown in Figure 3a. The 100 μm particles had the warmest *T*_50_ at −22.8 °C, followed by 2 μm at −23.2 °C, 10 μm at −23.4 °C, 1 μm at −23.5 °C, 6 μm at −23.7 °C, and 0.5 μm at −23.9 °C. While no significant differences were found between freezing temperature and particle size, the ice-active site density by mass, *n*_m_, increased with a decrease in particle size (Figure 3b).

**Figure 3:**
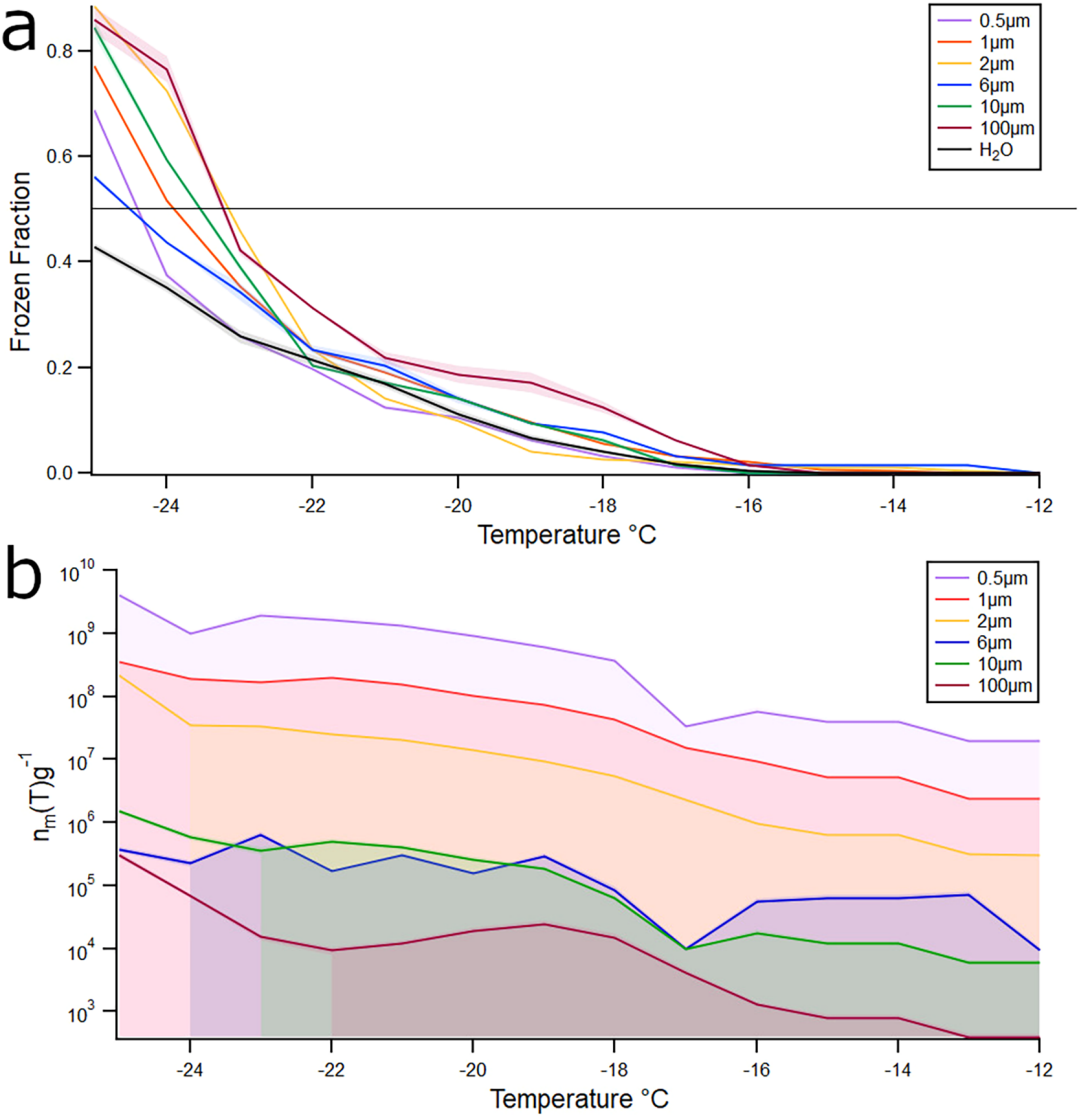
Impact of polymer size on ice nucleation. (a) Frozen fraction *F*(*T*) of aged polystyrene (PS) microplastics of varying sizes shown on the *y*-axis with freezing temperature on the *x*-axis. Water blank is depicted as a black line. (b) The calculated number of surface-active sites per unit mass, *n*_m_(*T*), for aged PS microplastics as a function of particle size, with *n*_m_(*T*) on the *y*-axis and freezing temperature on the *x*-axis. Shaded regions represent standard error, *n* = 96. At warmer temperatures (>−20 °C), uncertainty is inherently high and should be interpreted cautiously, as large errors reflect the freezing of only a few droplets, producing high variability; in some cases, values were constrained to a small positive lower bound when freezing could not be distinguished from background. At colder temperatures (<−22 °C), more droplets froze and replicate measurements converged, leading to smaller errors and smoother curves.^19^ Accordingly, treatment comparisons are most robust in the colder portion of the measured temperature range.

### Freezing of Microplastics with a Biofilm

Accumulation of biofilms on the surface of MPs was estimated using CFU counts after sonication. The *P. syringae* ice− strain was more abundant on PS, PSUV, and PEUV compared to the ice+ strain by a factor of 10 (Figure S4). PSUV was found to harbor the most ice+ bacteria based on CFU enumeration and had the highest median freezing temperature of −5.5 °C. However, CFU numbers alone were not a clear indicator of higher median freezing temperatures as the other polymers did not follow this trend (Figure S5).

To evaluate whether biofilm colonization translated to measurable changes in ice nucleation activity, we compared freezing temperatures of colonized and uncolonized MPs. As expected, the MPs colonized with wildtype ice+ *P. syringae* showed a statistically significant increase in the freezing temperatures compared to MPs without the ice+ strain (Figure 4a,b). Surprisingly, the ice+ biofilm on MPs had a median freezing temperature greater than that of 10^5^ cells/mL of bacteria (the average amount of cells removed from MPs, Figure S6) when immersed in water alone. The colonization of strictly the mutant ice− *P. syringae* strain had a general trend of increasing the median freezing temperature by approximately 1 to 2 °C, but only the ice− strain colonization of pristine PS was found to be statistically significant (*p* = 0.015) raising the median freezing temperature from −23 °C to −21 °C (Figure 4a,b). However, when comparing the ice− biofilm MPs and 10^6^ per mL ice− cells alone (average removed from MPs, Figure S3) there was a significant increase in median freezing temperatures (*p* < 0.0001).

**Figure 4:**
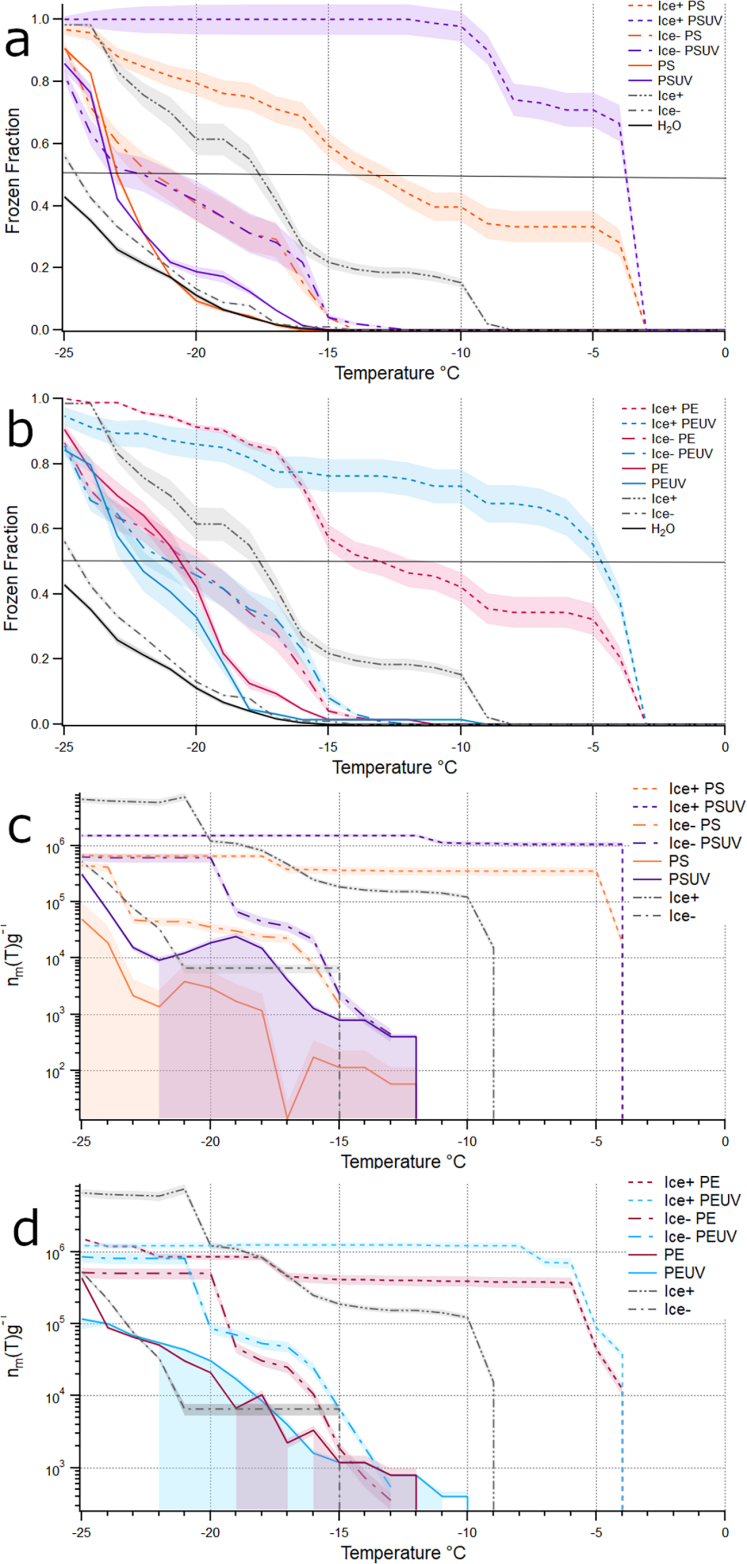
Impact of *P. syringae* wild type (ice+) and mutant (ice−) biofilm’s impacts on MPs as INPs. (a) Frozen fractions *F*(*T*) of ice+ and ice− cultured polystyrene (PS) and UV-treated PS (PSUV), (b) *F*(*T*) of ice+ and ice− cultured polyethylene (PE) and UV-treated PE (PEUV), (c) *n*_m_ of ice+ and ice− cultured PS and PSUV, and (d) *n*_m_ of ice+ and ice− cultured PE and PEUV. *P. syringae* (ice+ and ice−) without MPs are shown in gray, solid lines represent MP without a biofilm, and dashed lines represent those with a biofilm. Shading indicates standard error, *n* = 96. Uncertainty is greatest at warmer temperatures due to low frozen-droplet counts, while treatment comparisons are most robust at colder temperatures where replicate measurements converge.

Both ice+ and ice− *P. syringae* strains increased the number of active nucleation sites available at warmer temperatures, therefore increasing MPs’ INA (Figure 4c,d). The vast majority of microorganisms are unculturable in laboratory settings, and limited studies have tried to quantify the amount of biological ice nucleators present in nature, with many only selecting for *Pseudomonas* strains.^39,42–47^ To better represent a natural biofilm containing both ice+ and ice− bacteria, we averaged the INA data from the two strains. This combined data mean is shown in Figure 5. The average colonization of ice+ and ice− strains on MPs resulted in a statistically significant increase in median freezing temperature (*p* < 0.0001). A summary of all median freezing temperatures can be found in Table 2.

**Figure 5:**
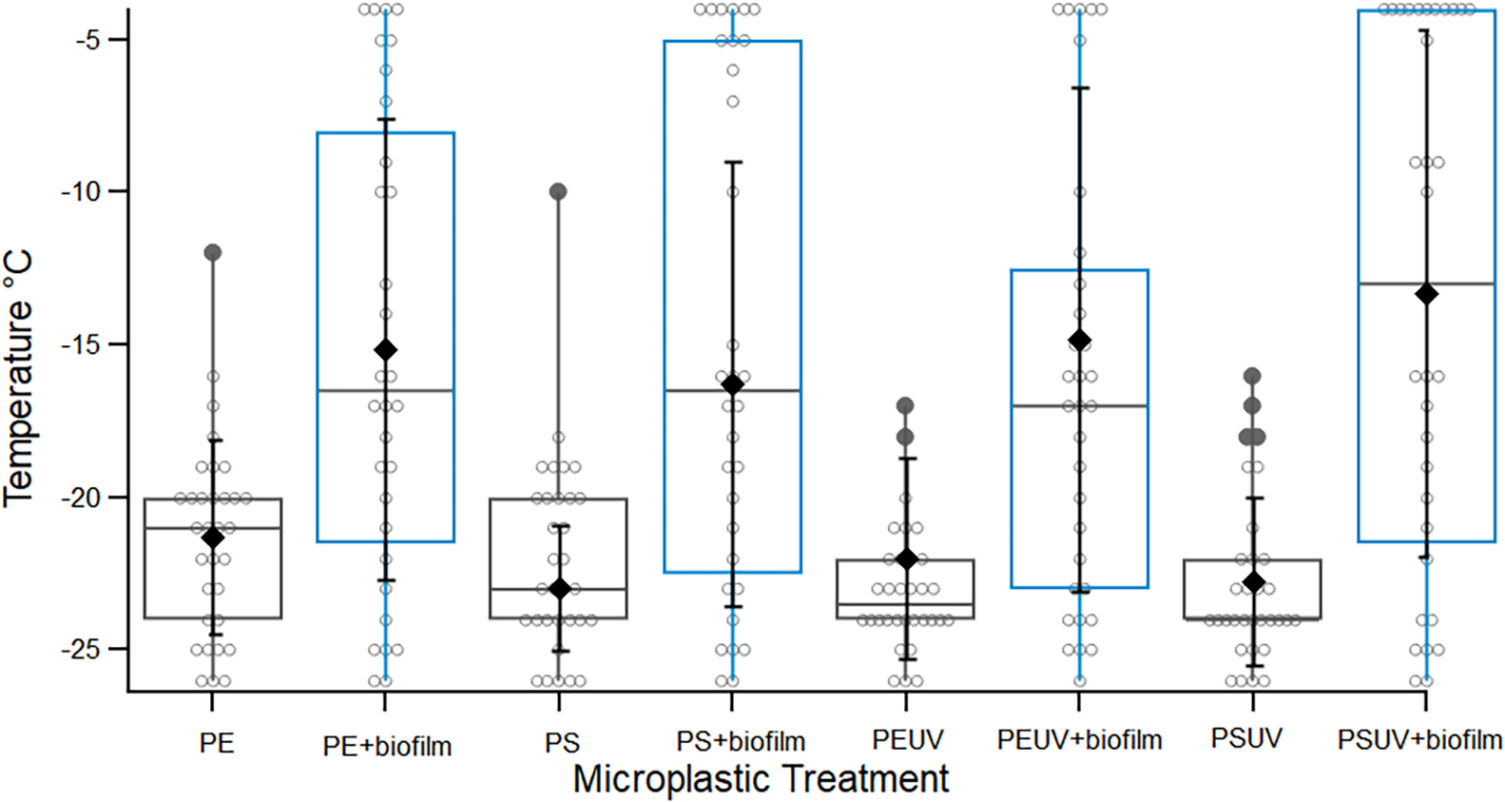
Boxplot showing the change in freezing temperatures between MPs with (blue) and without (dark gray) mean biofilm formation. Each boxplot includes data from at least three replicates. The box bounds indicate the interquartile range (25th–75th percentiles), with the median freezing temperature (*T*_50_) shown as a gray horizontal line. Filled circles represent outliers, and filled diamonds in black indicate mean with respective error bars. Whiskers extend to the 10th and 90th percentiles.

**Table 2:** Summary of Median Freezing Temperatures (*T*_50_) for all Microplastic Treatments^a^

sample	pristine *T*_50_ (°C)	aged *T*_50_ (°C)	biofilm *T*_50_ (°C)	Δ*T*_50_ (°C)
PS 0.5 μm	–23.78 ± 2.4	–23.91 ± 2.5	--	–0.13
AmPS 1 μm	–23.72 ± 3.5	–23.38 ± 3.0	--	+0.34
SuPS 1 μm	–23.72 ± 2.9	–23.43 ± 2.5	--	+0.29
CaPS 1 μm	–23.63 ± 2.9	–23.59 ± 2.6	--	+0.04
PS Bio 2 μm	–23.44 ± 2.8	–22.94 ± 2.5	--	+0.5
PS Strept 2 μm	–23.59 ± 3.4	–23.50 ± 2.2	--	+0.09
PS 6 μm	--	–23.69 ± 3.1	--	--
PS 10 μm	--	–23.41 ± 2.4	--	--
PS 100 μm	–23.0 ± 2.1	–22.78 ± 2.7	–16.31 ± 7.2	+0.22; +6.69
PE 100 μm	–21.31 ± 3.2	–22.03 ± 3.3	–15.19 ± 7.5	–0.72; +6.12

a Values represent the median temperature at which 50% of droplets froze (*T*_50_) for each microplastic type and treatment condition. Biofilm *T*_50_ represents the average freezing temperature of MPs colonized by both ice+ and ice− *P. syringae* strains on pristine polymers. Δ*T*_50_ indicates the change in freezing temperature between untreated and treated samples. Each value is based on at least three replicates. Abbreviations for polymer types and surface modifications are defined in Table 1 (e.g., AmPS = amine modified PS; SuPS = sulfate modified PS).

## Discussion

The discovery that biofilm-coated MPs exhibit altered ice nucleation activity fundamentally broadens our understanding of the environmental and climatic roles of MPs. Once considered inert environmental pollutants, our study displays MPs as dynamic substrates that not only harbor microbial life but actively shape the physical processes that govern cloud formation and precipitation. Biofilms on the surfaces of MPs actively modify physiochemical properties of MPs, underscoring the complex interplay between pollution, microbial ecology, and climate science. By connecting these domains, our findings not only deepen the understanding of MP impacts but also illuminate their role in climate feedback loops.

Microplastics of varying size, shape, and polymer type can act as ice nucleators and MP freezing results from this study generally align with previous findings.^13,18–20^ These studies, along with ours, illustrate that MPs in suspension induce a positive shift in freezing temperature when compared to pure water. Although the studies share this general trend, there are conflicting results regarding how aging impacts the propensity of MPs to act as INPs. Some studies, including our own, found that various forms of aging (e.g., UV, high temperatures, pressure, sulfuric acid, ammonium acid, and ozone) have little to no effect on ice nucleating activity compared to pristine MPs,^13,18,19^ though each study reported at least one outlier. In contrast, another study found that environmental stress can significantly enhance the ice nucleation activity of pristine MPs.^20^ These differing results are likely due to variations in the type of aging techniques as well as differences in MP source, polymer type, and particle shape.

We observed no significant effect of MP size on INA, a result consistent with the findings of Busse et al.^19^ However, our results differed when quantifying the active site density per mass of MPs. While Busse et al. did not observe a clear trend, we found that as MP size decreased, the number of available active sites increased (Figure 3b). Our findings support classical ice nucleation theory, which predicts that active site density scales with the surface area-to-volume ratio of particles.^48^

Ice nucleating bacteria may be more effective ice nucleators when residing on the surface of MPs. To our knowledge, Teska et al. were the first to report that biofilms residing on the surface of MPs can significantly alter their INA.^21^ However, there was no comparison between the INA of biofilms on MPs and that of bacteria alone in suspension. Our study found that the average number of ice+ *P. syringae* cells recovered from the MP surfaces on their own had a *T*_50_ lower than that of MPs with the same number of cells attached (Figure S6a). This suggests that ice+ bacteria may be more effective as ice nucleators when attached to MP surfaces than when suspended in water. Similarly, we also observed that the average number of ice− *P. syringae* cells recovered from the MP surfaces in suspension alone also had a *T*_50_ lower than that of MPs with the same number of cells attached (Figure S6b). This finding suggests that surface association enhances ice nucleation efficiency even for cells lacking intrinsic ice-nucleating activity. These results are consistent with the idea that surface-mediated organization or confinement of cells may facilitate ice nucleation processes.^49^ When *P. syringae* adheres to a MP surface, multiple synergistic mechanisms may potentially elevate its ice nucleation activity beyond what is observed in free suspension. Biophysically, the solid support and clustering of cells create a larger effective ice-nucleating surface, consistent with classical theory that larger nuclei catalyze freezing at warmer temperatures.^50^ Biologically, surface-induced stresses, such as nutrient limitation, low temperature, and biofilm signaling, enhance the production and organization of ice-nucleating proteins on the cell surface, ensuring that each attached cell presents its full ice-nucleating potential.^51^ The microplastic’s own properties—hydrophobic domains, rough texture, and surface charge—further modulate local water structure and nucleation kinetics. Together, these factors reveal a previously unrecognized synergy that makes a bacterium–microplastic complex an especially efficient ice nucleus.

This study is the first to report on the ability of biological non-ice nucleators to alter MP INA. While our MPs vs ice− biofilm MPs findings were not deemed statistically significant for PE, PEUV, and PSUV, the presence of the ice−*P. syringae* strain increased median freezing temperatures by 1 to 2 °C. However, the colonization of ice− *P. syringae* strain on PS did significantly increase median freezing temperature (*p* = 0.015). Notably, ice− had a higher number of cells present on PS, indicating that substantial biofilm formation can impact and ultimately enhance the ice nucleating activity of MPs (Figure S3). Markedly, as previously stated, when comparing the cells alone in suspension to the cells on the surface of MPs, there was a significant shift toward warming freezing temperatures for all polymers. Due to their size, most bacteria have the potential to act as a precursor to nucleation when present in high concentrations.^52^ Furthermore, it was recently discovered through ab initio calculations that when bacteria interact with inorganic material, the resulting H-bonds can lead to easier and more stable nucleation.^53^ Overall, this suggests that bacteria bonded to inorganic surfaces can initiate heterogeneous nucleation.

MPs with a biofilm on their surface are visualized in comparison to other known ice nucleators,^18–21,48,54–77^ including pristine and aged MPs in Figure 6. Colonized MPs exhibit a freezing range similar to that of volcanic ash,^73–75^ mineral dust,^69–72^ and sea spray,^48,68^ while also overlapping with biofilms on clothing textiles.^21^ This highlights the need for further investigation into the ice nucleation potential of diverse MP polymers and shapes, and how both organic and inorganic surface coatings may influence their activity.

**Figure 6:**
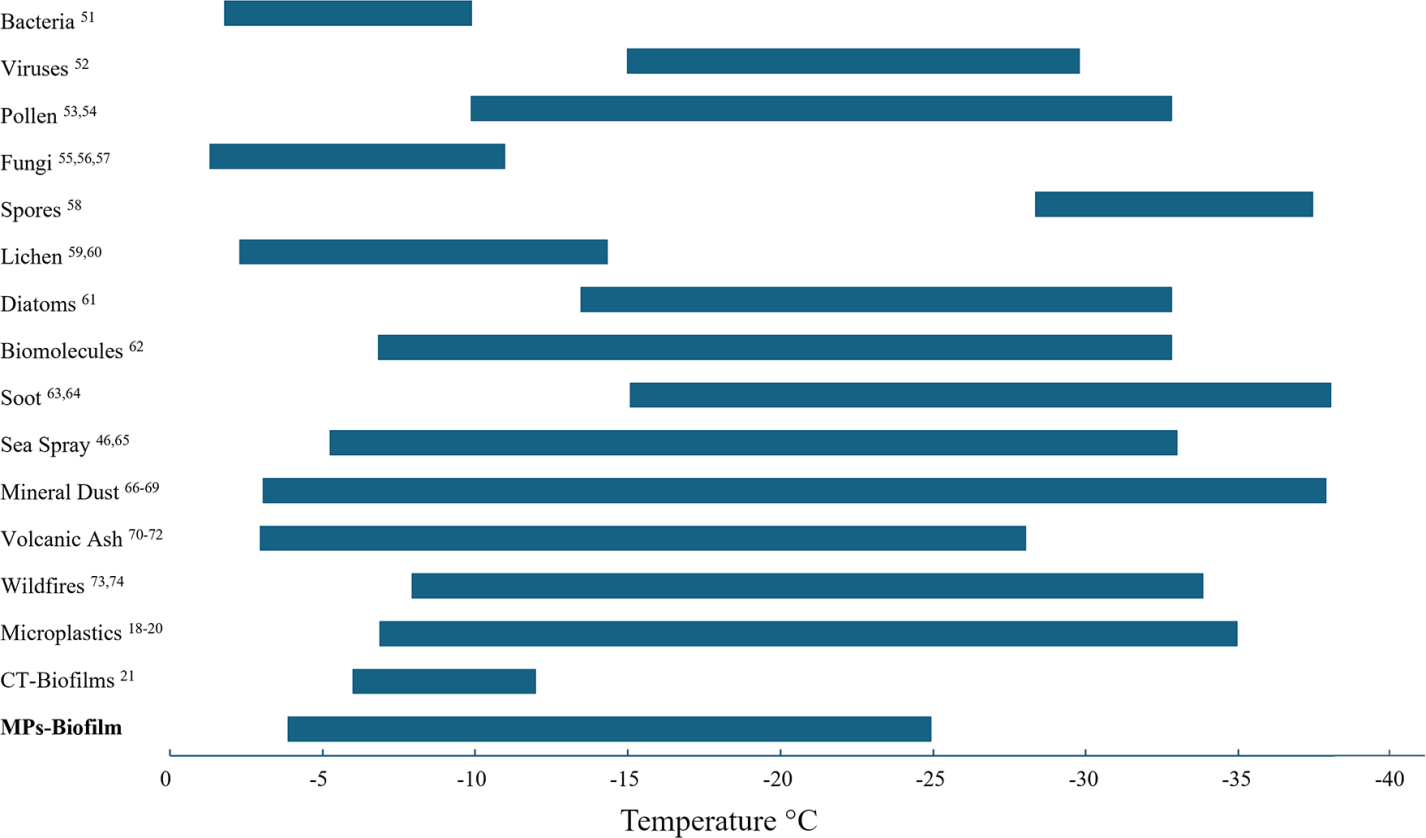
Common categories of ice nucleators and their reported range of ice formation temperatures. Due to differences in experimental methods, normalization approaches, and temperature scanning rates, this comparison is qualitative in nature.

While MP concentrations in the atmosphere are likely far less than the concentrations tested herein, laboratory loadings were intentionally elevated to ensure reproducible particle inclusion in each droplet. Reported MP concentrations in atmospheric liquids span several orders of magnitude depending on region and deposition type, ranging from 0 to 30 MPs/L in Antarctic snow,^10^ 10^3^–10^5^ MPs/L in European snow,^111^ 10-1154 MPs/L in cloudwater,^7,8^ and 140–460 MPs/L in rainwater.^12^ Rather than a direct simulation of typical atmospheric loadings, our high particle loading represents a mechanistic, upper bound whose results provide insight for regions with high atmospheric MP deposition or areas where INPs are limited, such as the Southern Ocean, especially within mixed-phase cloud regimes.^15,78–80^ INP-limited regions are particularly sensitive to changes in INP concentrations and accurately representing them can help reduce biases in climate models.^15,16,80^ Currently mineral dust dominates INP concentrations across the globe,^81^ but it has been suggested that—due to its higher density and lower aspect ratio—mineral dust has a shorter atmospheric lifetime than MPs.^16^ This implies that MPs may have a greater likelihood of influencing cloud microphysics in INP-limited regions. Our results along with those of others^18–21^ provide evidence that MPs can act as heterogeneous ice nucleators, suggesting a potential role in mixed-phase cloud formation. Accurately representing the number and activity of INPs on a global scale is crucial to better inform climate models, as mixed-phase clouds play a critical role in precipitation, energy balance, and global warming of the climate system.^16,82^

A key parameter that is still missing to inform models is an understanding of how much organic or inorganic material is present on the surface of MPs in the atmosphere. A plethora of literature exists documenting the plastisphere—the microbial communities that colonize the surface of MPs—in terrestrial and aquatic environments^23,83–87^ and many studies have shown that MPs adsorb various other pollutants onto their surface.^88–94^ It is likely that many MPs transported throughout the atmosphere have either, or both, organic and inorganic materials on their surfaces. Beyond shaping transport pathways, these additional ‘impurities’ on MP surfaces may alter both microbial colonization and ice nucleation potential.^95^ When secondary contaminants accumulate on MP surfaces, they can inherently alter surface properties such as hydrophobicity, charge, and roughness.^94^ Surface properties are key determinants of microbial attachment and biofilm stability.^96^ Modifying surface properties may not only change the composition of colonizing microbial communities but may also influence ice nucleation potential by modifying accessible nucleation sites or mediating interactions between MPs and microbial INPs. Coatings that mask surface-active sites may suppress freezing, whereas coatings that enhance biofilm anchoring or create nanoscale roughness may increase the number of accessible nucleation sites.^74,95,97^ Furthermore, contaminant aging could either enhance or diminish INA depending on the secondary contaminant composition, highlighting a critical need to evaluate environmentally conditioned MPs rather than pristine particles alone.^18,98,99^ Currently the body of literature on atmospheric microplastics has not preserved these attributes. Instead, MPs are typically subjected to a peroxide digestion, which removes any organic material from their surface, allowing for identification via various spectroscopic methods.^6–8,10,11,100^ Therefore, future studies should aim to retain subsets of collected atmospheric MPs for further investigation into their surface constituents. Accurately representing these components will help reduce biases in INP modeling and improve atmospheric transport models.

Beyond reducing model uncertainty, understanding what organisms reside on the surface of airborne MPs may also offer insight into disease tracking and monitoring. MPs collected from various environments have been shown to harbor pathogenic bacteria as well as a high abundance of antibiotic resistance genes (ARGs).^24,32,85,101–108^ However, it remains unknown how atmospheric MP biofilms compare to those in other environments in terms of pathogens and ARG content. This further highlights the need to investigate microbial communities on MPs collected directly from the atmosphere.

To our knowledge, this study provides the first comparison of ice nucleation behavior for MPs with and without biofilms. However, several limitations should be considered when extending these findings. First, freezing was monitored only to −25 °C due to instrumental constraints, capturing the onset and median portions of the freezing spectrum but not nucleation at colder temperatures. As a result, T_50_ values for weakly ice− nucleating samples may represent upper bounds rather than absolute medians, and differences among weaker ice nucleators may be underestimated. Accordingly, the results are most relevant to warmer mixed-phase cloud regimes, where ice nucleation occurs at relatively high subzero temperatures, rather than to colder cirrus cloud environments. Extending measurements to lower temperatures (e.g., −40 °C) would provide a more complete view of freezing behavior. Second, we used spherical PS and PE microspheres to control surface area and ensure reproducible biofilm attachment. While environmental MPs are typically irregular and roughened^1,6^ and should be studied, incorporating such particles would introduce variable surface areas that compromise comparisons among treatments. Third, environmentally relevant concentrations of airborne MPs are extremely low,^7,11,109^ and higher particle loadings were used here to ensure reproducible particle inclusion within droplets. This approach prioritizes mechanistic insight, and future work should evaluate whether similar effects occur under more dilute, atmosphere-like conditions. Fourth, UVC irradiation was used as an accelerated aging treatment to induce surface oxidation on laboratory time scales; this represents an extreme scenario and does not replicate natural atmospheric exposure dominated by UV-A and UV-B wavelengths. Finally, the irregular three-dimensional structure of biofilms prevents precise quantification of added surface area, restricting surface-area normalization to pristine particles only; advances in 3D imaging could improve this comparison in future studies. Despite these constraints, our results reveal clear biofilm-driven modifications to MP ice nucleation and provide a basis for future atmospheric studies.

As plastic production continues to rise globally, the atmospheric burden of MPs is also expected to increase. Combined with the accelerating effects of climate change, this will likely exacerbate plastic degradation and further elevate the release and atmospheric transport of MPs.^110^ This study contributes to growing evidence that MPs, across a range of polymer types and sizes, can act as heterogeneous ice nucleating particles under immersion freezing conditions.^18–21^ Notably, we show that biofilm colonization enhances their INA, particularly at warmer temperatures, by increasing the number of active nucleation sites.

These findings underscore the importance of incorporating environmentally relevant MPs, including those with organic coatings or microbial communities, in future laboratory assays and atmospheric INP models. Understanding the atmospheric role of MPs is still in its early stages. By demonstrating how surface-associated biology modifies their ice nucleation potential, this work helps bridge key gaps between MP occurrences in the environment and their possible climate impacts.^5,7,110^ Continued research is needed to characterize the vast diversity of MPs, across polymers, shapes, and surface chemistries, as well as the complex and dynamic biofilm communities they host, to better understand how these evolving materials interact with cloud microphysical processes.

## Supplementary Material



## Data Availability

Data underlying this manuscript are made accessible through the Virginia Tech Data Repository at https://doi.org/10.7294/29340182.

## Notes

Supplemental Document including additional experimental details, methods, results, and photographs of experimental setup (PDF)

## Abbreviations

MP: microplastic
INP: ice-nucleating particle
INA: ice nucleation activity
PS: polystyrene
PE: polyethylene
SEM: scanning electron microscopy
CFU: colony forming units
UV: ultraviolet
ARGs: antibiotic resistance genes
DI: deionized
