# Supplementary material for: Colonization of Microplastics by Different Strains of Pseudomonas Syringae Increases Ice-Nucleation Activity
Source: Environ Sci Technol. 2026 Mar 11;60(12):9438–50. doi: 10.1021/acs.est.5c18769 (PMC13045015; doi:10.1021/acs.est.5c18769)
Supplement: Supplementary file 1 [file es5c18769_si_001.pdf]

Supplementary Information for:

Colonization of Microplastics by Different Strains of *Pseudomonas syringae* Increases Ice-Nucleation Activity

Carrie Carpenter[1], Kelsey Kern[2], Regina Hanlon[1], Boris A. Vinatzer[1], David G. Schmale[1], Hosein Foroutan[3,]*

[1]School of Plant and Environmental Sciences, Virginia Tech, Blacksburg, VA 24061, USA
[2]Department of Biological Sciences, Virginia Tech, Blacksburg, VA 24061, USA
[3]Department of Civil and Environmental Engineering, Virginia Tech, Blacksburg, VA 24061, USA

*Corresponding Author: Hosein Foroutan, hosein@vt.edu

The following six page document contains additional methods, experiments, and results not present in the main text, including six additional figures and one table.

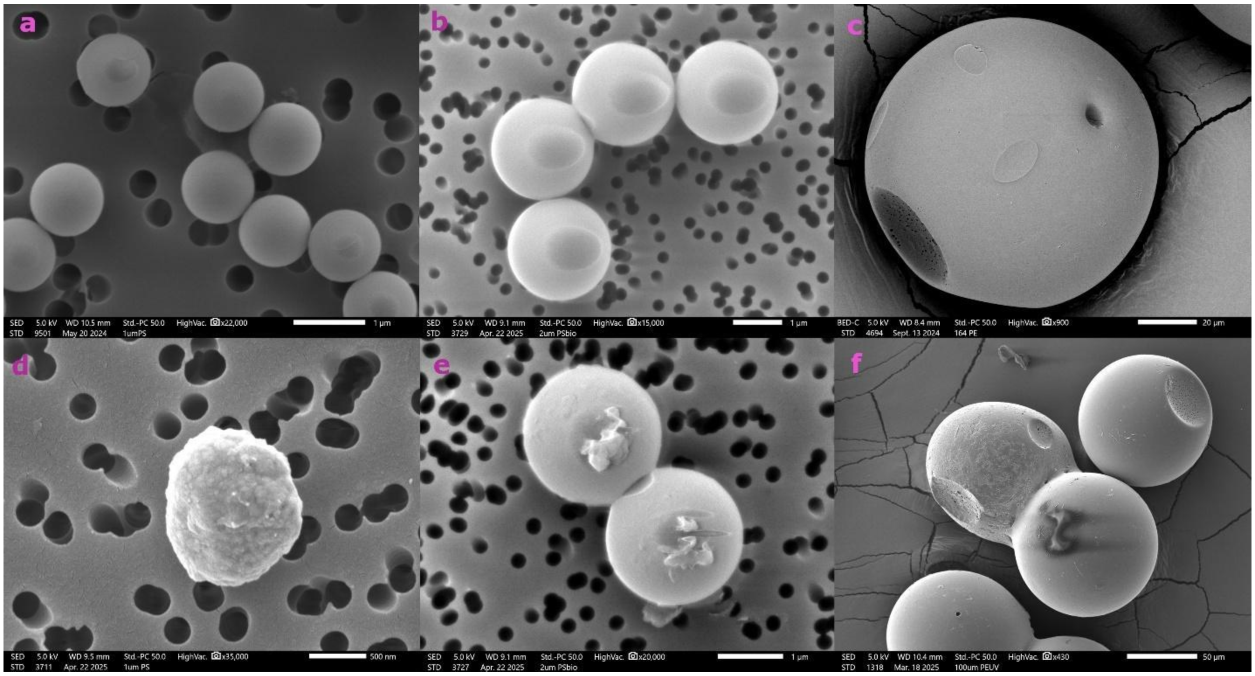

Figure S1. Scanning electron microscope (SEM) images of pristine microplastics (MPs) (a–c) and same MPs after hydrothermal aging (d, e) and UV aging (f) respectively. Image a/d is 1μm SuPS, b/e is 2μm biotin PS, and c/f is 100μm PE.

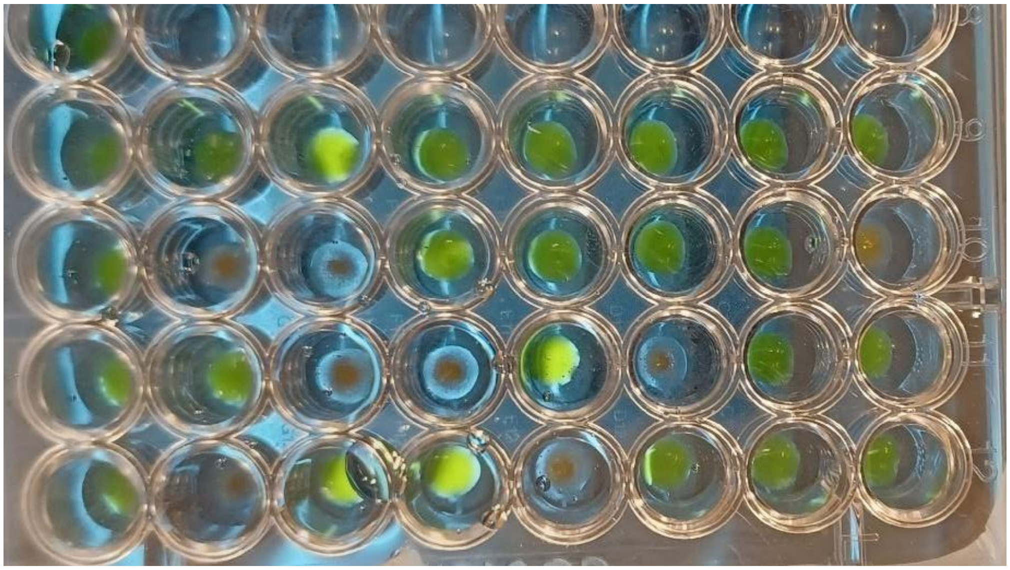

Figure S2. Images showing the color change of water from liquid (green) to frozen (orange) state. Example shown is 2 μm BioPS aged at -15°C.

We evaluated whether fluorescein affected droplet freezing in the absence and presence of MPs (pristine and aged). Two comparisons gave marginal p-values (~0.0496), but these did not remain significant after correcting for multiple comparisons (adjusted $\alpha = 0.017$). Aged MPs showed no effect of fluorescein ($p = 0.889$). Overall, fluorescein did not exert a consistent or systematic influence on freezing indicating that its use as a tracer did not affect the results.

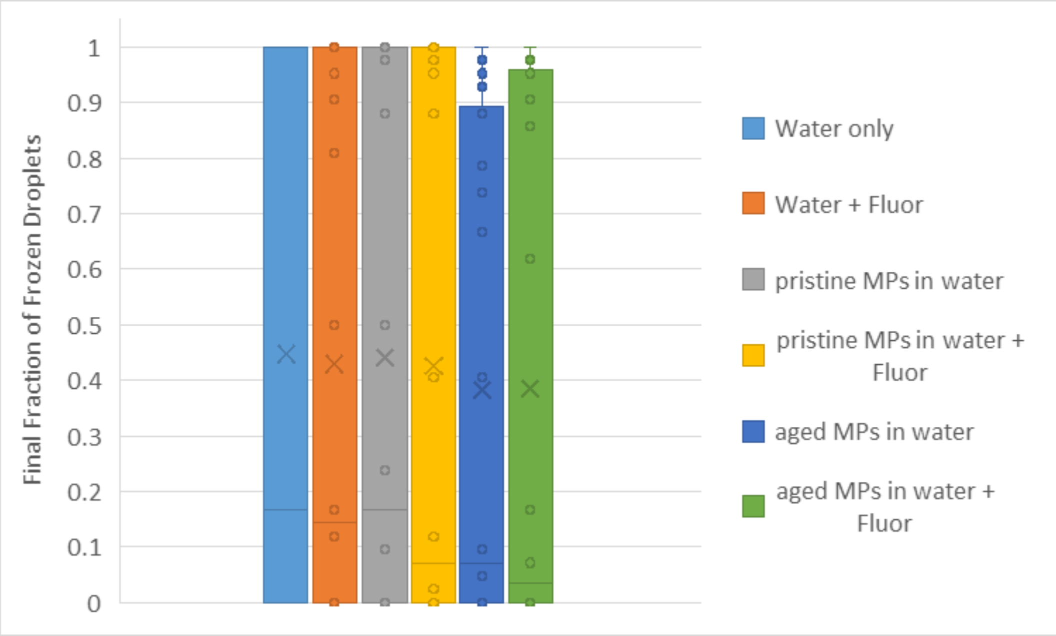

Figure S3: Final fraction of droplets frozen at -25°C for each treatment (n = 32 droplets per treatment). Colored bars represent each treatment. Individual circular markers indicate single-droplet outcomes, illustrating variability within each condition. The horizontal line within each bar denotes the median final fraction of frozen droplets, while the "×" symbol indicates the mean. Error bars, where present, represent the interquartile range (25th–75th percentile). All droplets froze completely by the end of the experiment; therefore, no variability (SE = 0) is shown.

Table S1. Change in number of active nucleation sites after aging treatments for each temperature. Temperatures above -10°C had no change.

Temperature °C	0.5 µm PS	1.0 µm SuPS	1.0 µm CaPS	1.0 µm AmPS	2.0 µm PS_Bio	2.0 µm PS_Strept	100 µm PS	100 µm PE
-10	0	0	0	$2x10^5$	0	0	0	$-4x10^2$
-11	0	0	0	$2x10^5$	0	0	0	$-4x10^2$
-12	0	0	0	$2x10^5$	0	0	0	0
-13	0	0	0	$2x10^5$	$-2x10^4$	0	0	0
-14	0	0	0	$1x10^7$	$2x10^6$	$2x10^6$	0	0
-15	0	$9x10^4$	$1x10^6$	$1x10^7$	$2x10^6$	$2x10^6$	0	0
-16	0	0	$-4x10^6$	$6x10^6$	$8x10^5$	$1x10^6$	$-1x10^2$	$2x10^3$
-17	$1x10^8$	$-2x10^6$	$-2x10^7$	$2x10^7$	$-2x10^6$	$-2x10^6$	$-4x10^3$	$-2x10^3$
-18	$-2x10^6$	$-4x10^7$	$2x10^6$	$-4x10^7$	$-2x10^5$	$-5x10^6$	$-8x10^3$	$-1x10^4$
-19	$-4x10^6$	$2x10^6$	$-2x10^5$	$-5x10^7$	$-6x10^5$	$-3x10^5$	$-1x10^4$	$-1x10^4$
-20	$-8x10^7$	$10x10^6$	$-9x10^7$	$-9x10^7$	$-1x10^6$	$-6x10^5$	$-2x10^3$	$-10x10^3$
-21	0	$1x10^7$	$-1x10^7$	$-1x10^8$	$-2x10^6$	$-1x10^6$	$2x10^4$	$-1x10^4$
-22	0	$1x10^7$	$-2x10^7$	0	$-2x10^6$	$-1x10^6$	0	$-3x10^3$
-23	0	$4x10^6$	$2x10^8$	$2x10^7$	$-1x10^7$	$1x10^6$	$8x10^3$	$-3x10^3$
-24	$2x10^9$	$9x10^6$	$3x10^8$	$-10x10^7$	$-7x10^6$	$-2x10^7$	$7x10^4$	$-1x10^4$
-25	$2x10^8$	$2x10^8$	$-7x10^7$	$-4x10^8$	$-3x10^8$	$-10x10^7$	$-4x10^3$	$3x10^5$

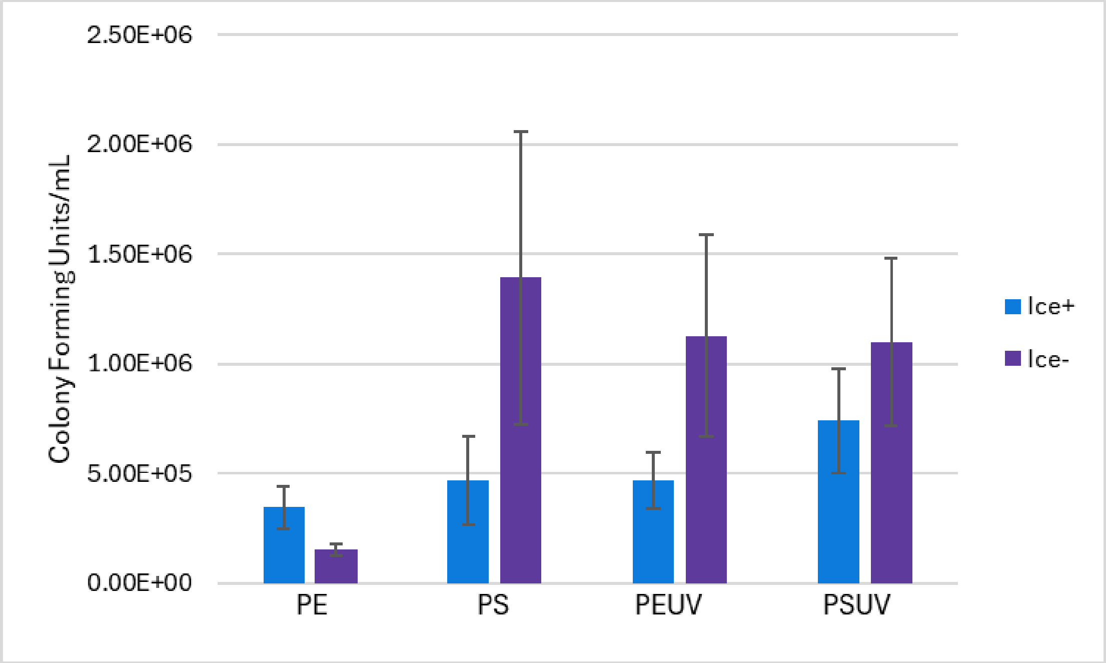

Figure S4. Average colony-forming units (CFU) of *P. syringae* ice+ and ice- strains recovered from each of the 100 µm polymer types.

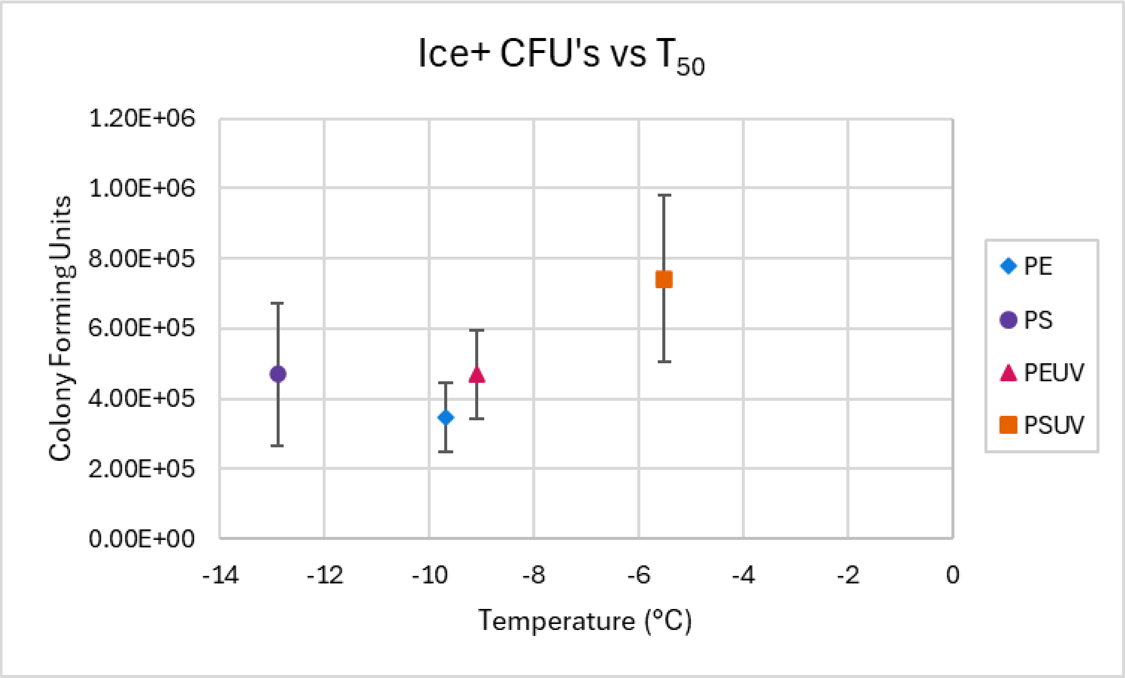

Figure S5. Relationship between average colony-forming units (CFUs) recovered from each polymer (y-axis) and their corresponding median freezing temperature T_{50} (x-axis).

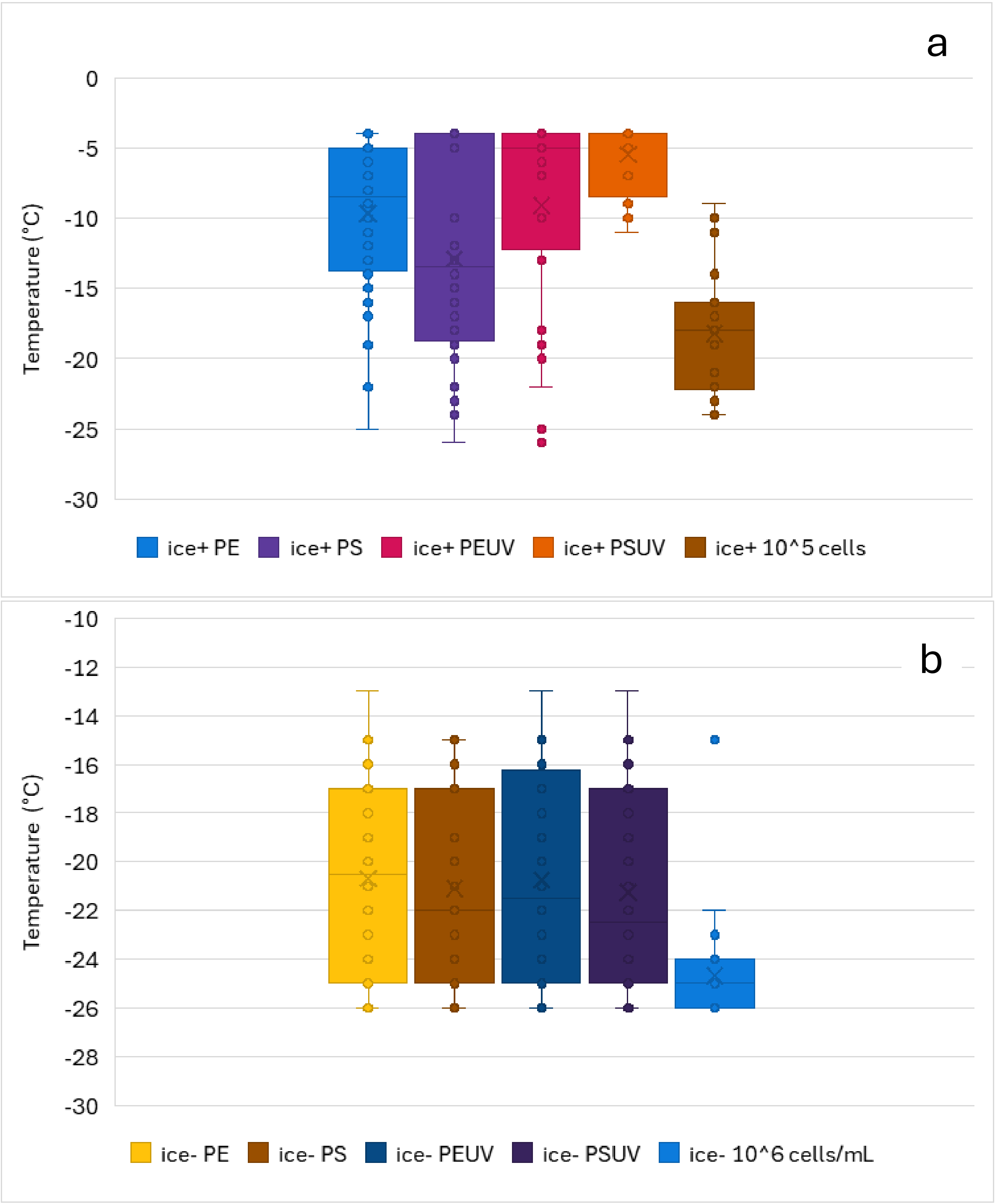

Figure S6. Boxplots showing the difference in freezing temperatures between MPs colonized with ice+/- biofilm and the cells alone. a) Ice+ biofilm MPs vs suspended ice+ cells at a concentration of 10^5 cells/mL . b) Ice- biofilm MPs vs suspended ice- cells at a concentration of 10^6 cells/mL. Freezing temperature is shown on the y-axis. Boxes and whiskers represent the same statistical parameters as described in Figure 2.
